# Bioequivalence study of testosterone undecanoate soft capsules in healthy postmenopausal women under fed conditions: a single-center, four-period, repeated crossover trial

**DOI:** 10.3389/fphar.2026.1763835

**Published:** 2026-03-03

**Authors:** Nanxing Li, Fengjia Zhu, Zhongyuan Zhao, Shilong Zhu, Jinlian Wu, Xu Zuo, Jinjin Shi, Xiuhui Qian, Xiaoping Zhang, Yuanyuan Hu, Yueran Lv, Jing Chen, Tiandong Zhang

**Affiliations:** 1 Research and Development Center, Zhejiang Medicine Co., Ltd., Shaoxing, Zhejiang, China; 2 School of Engineering, China Pharmaceutical University, Nanjing, Jiangsu, China; 3 Department of Clinical Pharmacology, Xinxiang Central Hospital, Xinxiang, Henan, China; 4 Shanghai Xihua Scientific Co., Ltd., Shanghai, China

**Keywords:** bioequivalence, Chinese healthy participants, pharmacokinetics, safety, testosterone undecanoate

## Abstract

**Purpose:**

This study aimed to systematically evaluate the pharmacokinetics, bioequivalence, and safety of a single postprandial oral dose of testosterone undecanoate (TU) and its originator drug Andriol Testocaps^®^ in healthy postmenopausal Chinese women, providing theoretical support for optimizing hormone replacement therapy protocols.

**Methods:**

A randomized, open-label, two-treatment, four-period, single-center, single-dose crossover clinical trial was conducted at Xinxiang Central Hospital. Participants received single oral doses of 40 mg TU or Andriol Testocaps^®^ in each period. Serial blood samples were collected from 0 to 24 h post-dose.

**Results:**

The average adjusted geometric mean ratios (GMR) (90% CI) for the primary PK parameters C_max_, AUC_0-t_, and ${\text{AUC}}_{0-\infty }$ were 102.20% (90.32%–115.63%), 99.85% (92.82%–107.41%), and 99.79% (92.90%–107.20%). All 90% CI for C_max_, AUC_0-t_, and ${\text{AUC}}_{0-\infty }$ fell within the 80%–125% bioequivalence range. The two drugs showed comparable results for the other PK parameters. These results indicate that the two drugs were bioequivalent.

**Conclusion:**

TU demonstrated bioequivalence to Andriol Testocaps^®^ under fed conditions in Chinese healthy participants, with comparable safety and tolerability profiles. These results advocate the clinical application of generic TU as a potential alternative to originator drug Andriol Testocaps^®^ in the treatment.

## Introduction

Late-Onset Hypogonadism (LOH) is a clinical syndrome closely associated with aging, characterized by serum testosterone (T) levels below the normal reference range for healthy adult males, accompanied by a series of clinical symptoms (Bhasin et al., 2010; Morelli et al., 2007). Given that testosterone deficiency is the direct pathophysiological basis of LOH, testosterone replacement therapy (TRT) has been established as the standard treatment for this condition, irrespective of fertility requirements (Nieschlag, 2015; Loeb et al., 2017). As a core drug in TRT, testosterone undecanoate (TU) exerts its clinical effects by externally supplementing testosterone to effectively alleviate low-testosterone-related symptoms in LOH patients, such as decreased libido, reduced muscle mass, fatigue, and mood disorders (Loeb et al., 2017; Klepsch et al., 1982). TU, an ester derivative of testosterone, functions through enzymatic hydrolysis by esterases *in vivo* to slowly release active testosterone, which is subsequently converted to dihydrotestosterone (DHT) via 5α-reductase in target tissues. It ultimately binds to androgen receptors, regulating gene expression and exerting physiological effects such as promoting protein synthesis and inhibiting protein catabolism (Poletti et al., 2001).

TU is available in diverse dosage forms, including oral and injectable preparations, each addressing different clinical needs: intramuscular suspensions are suitable for patients requiring long-term testosterone replacement therapy, with efficacy comparable to marketed injections and lower local irritation (Testosterone Undecanoate-Schering AG, 2004; Harle et al., 2005); transdermal formulations avoid first-pass effects through skin absorption, making them appropriate for patients intolerant to injections or oral administration (Wang et al., 2004; Hameed et al., 2003).

TU possess unique pharmaceutical properties: their lipid-soluble structure enables absorption via the intestinal lymphatic system, circumventing hepatic first-pass metabolism and thereby ensuring the effectiveness of oral administration (Horst et al., 1976). This characteristic positions them as a critical long-term treatment option for LOH patients.

Globally, TU oral preparations primarily include originator drugs and generics. In the Chinese market, the imported originator drug—TU soft capsules (Andriol Testocaps^®^, 40 mg)—manufactured by N.V. Organon and Catalent France Beinheim S.A.—has long been a key therapeutic choice for LOH patients. In December 2020, Merck & Co., Inc. (China) Investment Co., Ltd. discontinued the supply of this product in the Chinese market due to the global withdrawal plan of the parent company, with formal market exit occurring in January 2021. This led to an urgent clinical need for alternative, high-quality TU preparations to ensure treatment continuity. Following the delisting of Andriol Testocaps^®^ in China, domestic LOH treatment faced a sharp decline in short-term drug accessibility, necessitating the development of domestic generics to fill the market gap.

In accordance with the requirements of the National Medical Products Administration (NMPA), any generic drug must undergo re-evaluation of its quality equivalence to the originator drug before adopting new regulatory measures. This study aimed to evaluate and compare the pharmacokinetics (PK), bioequivalence (BE), and safety of a single postprandial dose of the test formulation versus the reference formulation in healthy postmenopausal Chinese female participants, adhering to relevant regulatory guidelines for bioequivalence trials.

## Methods

### Study materials

The test formulation, TU soft capsules, was supplied by manufactured by Zhejiang Medicine Co., Ltd. Xinchang Pharmaceutical Factory (Bath NO.: 1052202, 40 mg), while the reference formulation, Andriol Testocaps^®^, was provided by Catalent France Beinheim S.A. (Bath NO.: T023247, 40 mg). All study drugs were offered by Xinchang Pharmaceutical Factory.

### Study population and design

This clinical trial was carried out at the Clinical Trial Research Center of Xinxiang Central Hospital. According to the requirements of the sponsor, the clinical trial was registered at chinadrugtrials.org.cn (CTR20223150, registered on 14 December 2022), Which was an authoritative registration authority widely recognized under Chinese laws and regulations. The study protocol and its amendments met the Good Clinical Practice guidelines and Declaration of Helsinki. The study protocol was approved by the Ethics Committee of Xinxiang Central Hospital (approval number: 2022-075). All participants willingly consented to take part in this study and provided written informed consent.

The clinical trial recruited Chinese healthy naturally postmenopausal females who were in good health and aged between 45 and 65 years (inclusive), with cessation of menses for≥12 months, and the BMI range was 18–28 kg/m^2^ (inclusive). Postmenopausal women were chosen as surrogates for hypogonadal men due to their stable, low endogenous testosterone levels, which minimize confounding fluctuations inherent in male subjects. This selection aligns with regulatory guidance for endogenous compound bioequivalence studies. Female participants had a minimum weight of 45 kg and a total testosterone level ≤0.75 ng/mL. The participants underwent a comprehensive evaluation. Participants who satisfied the eligibility criteria were included, whereas those who fulfilled any of the exclusion criteria were not recruited. Additional details regarding the criteria for including and excluding individuals from the study can be found in the Supplementary Material.

Healthy participants were randomly assigned to either the T-R-T-R sequence group or the R-T-R-T sequence group in a 1:1 ratio. Participants received oral administration of 40 mg TU soft capsules (T/R) under postprandial conditions on Day 1/Day 6/Day 11/Day 16. On the first day of each dosing cycle, participants, after an overnight fast of at least 10 h, initiate the consumption of a high-fat meal (800–1000 calories) 30 min prior to medication administration. Subsequently, participants orally take 40 mg of Andriol Testocaps^®^ or TU according to the schedule. The washout period between periods was set to be no less than 5 days.

### Sample size determination

To compare the bioequivalence of TU and Andriol Testocaps^®^, a single-center, open-label, randomized, single-dose, four-period, fully repeated crossover design was conducted. The determination of the sample size was based on prior reports indicating that the intra-participant variability (CV_W%_) of testosterone undecanoate C_max_ and AUC ranged from 30% to 50% (Yin et al., 2012; Schnabel et al., 2007). This study employed a four-period fully replicated crossover design with pharmacokinetic parameters (AUC, C_max_) as primary analysis indices. Assuming α = 0.05, β = 0.2, Intra-CV = 30%, geometric mean ratio (GMR) of test/reference formulation = 0.90, and bioequivalence interval of 80.00%–125.00%, PASS software calculated a minimum sample size of 40 participants. Considering a certain dropout rate, 48 participants were ultimately enrolled in the postprandial trial.

### Blood sampling and bioanalytical assays

Blood samples were collected in each study period. For sample collection, vacuum collection tubes pre-cooled in an ice-water bath containing K_2_-ethylenediaminetetraacetic (EDTA-K_2_) anticoagulant were used, with blood drawn at 24 time points: 2.0 h before dosing (−2.0 h), 1.0h before dosing (−1.0 h), 0 h (dosing time), and 1.0 h, 2.0 h, 3.0 h, 3.5 h, 4.0 h, 4.5 h, 5.0 h, 5.5 h, 6.0 h, 6.5 h, 7.0 h, 7.5 h, 8.0 h, 9.0 h, 10.0 h, 11.0 h, 12.0 h, 13.0 h, 14.0 h, 16.0 h, 24.0 h after dosing. Biologic sample collection and processing were conducted under ice-bath white light conditions. The collected whole blood was centrifuged at 2 °C–8 °C with a relative centrifugal force (RCF) of 1700 g for 10 min to separate plasma. The resulting plasma supernatant was stored at −80 °C prior to analysis.

The plasma concentrations of Testosterone undecanoate and Testosterone were measured with a validated liquid chromatography with tandem mass spectrometry (LC-MS/MS) method developed by Shanghai Xihua Scientific Co. Ltd. Testosterone undecanoate, Testosterone and their internal standards (D_3_-Testosterone undecanoate and Testosterone-^13^C_3_) were isolated from human plasma using solid-phase extraction with C_18_ (Cleanert ODS C_18_, Agela). Elution was performed using acetonitrile. The eluate was evaporated to dryness, and the residue subsequently dissolved in methanol and water 60:40 (V:V). For calibration samples, the internal standard solution was vortexed with surrogate matrix and working solutions. The concentration range of the calibration curve used for sample analysis was 0.300–120 ng/mL for Testosterone undecanoate and 0.0750–15.0 ng/mL for Testosterone.

Bioanalysis was performed on a Shimadzu LC-30AD system with an Applied Biosystems Triple Quadrupole 6500+ mass spectrometer equipped with an APCI source. The data were recorded using the Analyst1.7.2 software (Applied Biosystems, USA). Chromatographic separation was achieved on an Poroshell 120 EC-C18 column (100 × 2.1 mm, 2.7 μm; Agilent). The mobile phase consisted ofwater with 0.1% formic acid and 2 mM ammonium acetate (A) and methanol (B), and the flow rate was 0.50 mL/min. Quantification was performed in positive ion mode through the multiple reaction monitoring of the ion pairs of 457.2/271.2 for Testosterone undecanoate, 460.2/274.2 for D_3_-Testosterone undecanoate, 289.2/97.2 for Testosterone and 292.2/100.2 for Testosterone-^13^C_3_.

### Safety evaluations

Vital signs (including auricular temperature, pulse rate, and sitting blood pressure) were measured at 1.0 h before dosing and at 2.0 ± 0.5, 6.0 ± 0.5, 12.0 ± 0.5, and 24.0 ± 1.0 h post-dosing in each study period. Researchers promptly documented adverse events (AEs) throughout the trial. For participants in Period 4, safety assessments (physical examination, vital sign reevaluation, 12-lead electrocardiogram, and laboratory tests) were conducted on the day of pharmacokinetic blood sampling completion. A telephone follow-up was performed 7 ± 2 days after study discharge to inquire about subsequent AEs and document their outcomes. All AEs observed during the trial were followed until resolution.

### Statistical analysis

Pharmacokinetic parameters of testosterone undecanoate—including C_max_ (maximum plasma concentration), AUC_0-t_, ${\text{AUC}}_{0-\infty }$ , t_max_, λ_z_ (elimination rate constant), t_1/2_, and AUC__%Extrap_—were calculated using Phoenix WinNonlin 8.3 software via non-compartmental pharmacokinetic analysis. Descriptive statistics were generated for all parameters using SAS 9.4. For test and reference formulations, the natural logarithm-transformed C_max_, AUC_0-t_, and ${\text{AUC}}_{0-\infty }$ values underwent mixed-effects model analysis of variance. GMRs of the two formulations were computed at the 90% confidence level, with ratios converted back to linear scale via antilogarithmic transformation. A statistical significance threshold of P < 0.05 was applied.

Equivalence criteria were evaluated based on reference formulation CV_w_%. If CV_w_% < 0.294 (i.e., CV_w_% < 30%), the average bioequivalence (ABE) method was applied, requiring the 90% confidence interval (CI) of GMR for C_max_, AUC_0-t_, and ${\text{AUC}}_{0-\infty }$ to fall within 80.00%–125.00%. For CV_w_% ≥ 30%, the reference-scaled average bioequivalence (RSABE) method was implemented, mandating simultaneous fulfillment of two conditions: (1) GMR upper confidence bound ≤ pre-specified upper limit (calculated via FDA-recommended formula) and (2) GMR point estimate within 80.00%–125.00%. Both testosterone undecanoate (primary analyte) and testosterone (supportive analyte) adhered to these equivalence criteria.

## Results

### Participants

A total of 127 participants were screened, and 48 participants (all female) were ultimately enrolled, including 47 Han Chinese and 1 Miao ethnic participant. The mean (±SD) age was 53.67 ± 3.33 years, with average height of 158.40 ± 5.16 cm, weight of 60.08 ± 6.07 kg, and body mass index (BMI) of 23.93 ± 1.89 kg/m^2^, as shown in Table 1. The study employed a four-period replicate crossover design with two treatment sequences: T-R-T-R and R-T-R-T, each comprising 24 participants. All enrolled participants completed the trial without premature withdrawal, adhering to the scheduled drug administration protocol.

**TABLE 1:** Baseline demographics characteristics.

Parameters	Mean ± SD
Age (years)	53.67 ± 3.33
Height (cm)	158.40 ± 5.16
Weight (kg)	60.08 ± 6.07
BMI (kg/m^2^)	23.93 ± 1.89

### Pharmacokinetic properties

**FIGURE 1:**
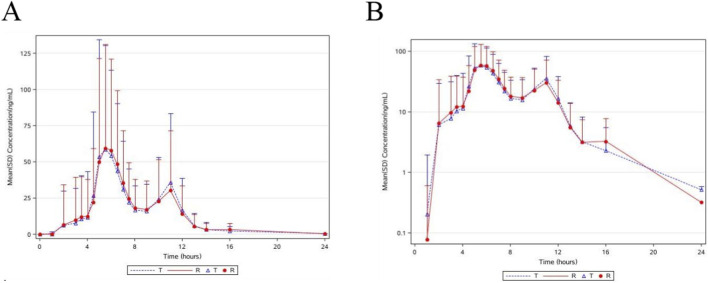
Mean plasma concentration-time profiles of testosterone undecanoate under linear scale (Mean ± SD) (PKCS) **(A)**; Mean plasma concentration-time profiles of testosterone undecanoate under semi-logarithmic scale (Mean ± SD) (PKCS) **(B)**. Note: Data are presented as means ± standard deviation. “T” denotes the test formulation administered in alternating period, while “R” specifies the reference formulation administered in complementary periods. The plotted values reflect the average plasma concentration across both periods for each formulation.

Figure 1 presents the mean plasma concentration-time profiles of testosterone undecanoate under postprandial conditions for both test and reference formulations. The corresponding pharmacokinetic parameters are summarized in Table 2. For testosterone undecanoate, the primary pharmacokinetic parameters (C_max_, AUC_0-t_, ${\text{AUC}}_{0-\infty }$ ) were analyzed using a mixed-effects model after natural logarithm transformation. The model incorporated the effects of dosing sequence, formulation factors, and dosing periods to compare their influences on parameter variability.

**TABLE 2:** The pharmacokinetic parameters (N = 95–96).

Pharmacokinetic parameters	T	R
T_max_^#^(h)	6.49 (1.9917, 13.9917)	6.49 (1.9919, 15.9917)
C_max_ (ng/mL)	113.39 ± 75.73 (66.78)	110.09 ± 67.59 (61.39)
AUC_0-t_ (h·ng/mL)	280.44 ± 129.92 (46.33)	279.48 ± 122.95 (43.99)
${\text{AUC}}_{0-\infty }$ (h·ng/mL)	285.26 ± 128.66 (45.10)	285.07 ± 120.40 (42.24)
λ_Z_ (1/h)	0.8116 ± 0.3395 (41.83)	0.8378 ± 0.3672 (43.82)
t_1/2_(h)	1.11 ± 0.79 (71.36)	1.08 ± 0.78 (72.34)
AUC__%Extrap_ (%)	1.53 ± 5.19 (339.67)	1.66 ± 5.16 (310.27)

Data are presented as mean ± SD (%CV), except for T_max_, which is expressed as median (minimum, maximum). For randomization number C010 (sequence R-T-R-T) in Period 1 and randomization number C028 (sequence T-R-T-R) in Period 3, fewer than 3 non-below-the-limit-of-quantification (non-BQL) sampling points were available following the C_max_ of testosterone undecanoate. Accordingly, the corresponding pharmacokinetic parameters λ_z_, t_1/2_, ${\text{AUC}}_{0-\infty }$ , and AUC__%Extrap_ for these periods were treated as missing.

The results indicated no statistically significant differences (P > 0.05) in C_max_, AUC_0-t_ and ${\text{AUC}}_{0-\infty }$ between the test and reference formulations for both dosing sequence and formulation factors. However, significant differences (P < 0.05) were observed across dosing periods. Detailed pharmacokinetic analyses of testosterone (baseline-corrected and non-corrected) are provided in Supplementary Material S1, S2.

### Assessment of bioequivalence

As shown in Table 3, the within-participant coefficient of variation (S_WR_) for the reference formulation’s C_max_ of testosterone undecanoate was 0.4801 (>0.294), indicating CV_W%_ of 50.91% (>30%). Therefore, reference-scaled average bioequivalence (RSABE) was applied for bioequivalence evaluation with a regulatory acceptance range of −0.1329 (<0). The geometric mean ratio (GMR) of C_max_ between the test and reference formulations was 102.20%, falling within the 80.00%–125.00% range.

**TABLE 3:** Summary of bioequivalence assessment.

​	ABE				​	RSABE				
Pharmacokinetic parameters	T	R	T/R ratio (%)	90% CI (%)	S_WR_	CV_w_ (%)	Acceptance criterion	T/R ratio (%)	Power%	Application methods
C_max_ (ng/mL)	92.50	90.51	102.20	90.32–115.63	0.4801	50.91	−0.1329	102.20	>99.99	RSABE
AUC_0-t_ (h·ng/mL)	254.23	254.61	99.85	92.82–107.41	0.2871	29.31	−0.0485	99.85	99.93	ABE
${\text{AUC}}_{0-\infty }$ (h·ng/mL)	259.62	260.15	99.79	92.90–107.20	0.2838	28.96	−0.0471	99.58	99.95	ABE

The acceptance criterion was defined as the upper bound of the one-sided 95% confidence interval. For participant C010 (sequence R-T-R-T) in Period 1 and participant C028 (sequence T-R-T-R) in Period 3, fewer than 3 non-below-the-limit-of-quantification (non-BQL) sampling points were available following the testosterone undecanoate C_max_. Consequently, the ${\text{AUC}}_{0-\infty }$ (area under the curve extrapolated to infinity) values for these periods were treated as missing data.

For the AUC_0-t_ and ${\text{AUC}}_{0-\infty }$ parameters of testosterone undecanoate, the S_WR_ values (0.2871 and 0.2838) were <0.294, corresponding to CV_W%_ values of 29.31% and 28.96% (<30%), respectively. Thus, average bioequivalence (ABE) criteria were applied. The GMRs and 90% confidence intervals for AUC_0-t_ and ${\text{AUC}}_{0-\infty }$ were 99.85% (92.82%–107.41%) and 99.79% (92.90%–107.20%), both within the 80.00%–125.00% range. Sensitivity analyses of testosterone undecanoate confirmed consistency with the primary analysis, supporting bioequivalence between the test and reference formulations (Supplementary Material S3). Bioequivalence was also demonstrated for both baseline-corrected and non-corrected testosterone levels (Supplementary Material S4, S5).

### Safety evaluations

As shown in the study, a total of 7 participants experienced 8 AEs with an incidence rate of 14.6% (7/48), all occurring during the test formulation period. Additionally, 5 participants reported 6 adverse drug reactions (ADRs) with an incidence rate of 10.4% (5/48), also exclusively during the test formulation administration phase. The most common AE was urine leukocyte positivity.

All adverse events were classified as Grade 1 severity, with no serious adverse events (SAEs), serious adverse drug reactions (SADRs), AEs/ADRs leading to withdrawal, or irreversible outcomes observed. Notably, all 8 AEs resolved spontaneously without sequelae under non-intervention conditions, demonstrating full reversibility. These findings collectively indicate comparable acute tolerability for both the test and reference formulations of testosterone undecanoate soft capsules when administered postprandially to healthy participants. Detailed AE summaries are provided in Table 4.

**TABLE 4:** Summary of AEs.

Adverse events	Test preparation (N = 48)		Reference reagent (N = 48)		Total (N = 48)	
SOC/PT	Number of cases	Number of cases (%)	Number of cases	Number of cases (%)	Number of cases	Number of cases (%)
Total	8	7 (14.6)	0	0 (0)	8	7 (14.6)
Various examinations	7	6 (12.5)	0	0 (0)	7	6 (12.5)
Positive urinary leukocytes	3	3 (6.3)	0	0 (0)	3	3 (6.3)
Elevated serum triglycerides	1	1 (2.1)	0	0 (0)	1	1 (2.1)
Decreased white blood cell count	1	1 (2.1)	0	0 (0)	1	1 (2.1)
Urinary sediment detected	1	1 (2.1)	0	0 (0)	1	1 (2.1)
Increased human chorionic gonadotropin	1	1 (2.1)	0	0 (0)	1	1 (2.1)
Gastrointestinal system diseases	1	1 (2.1)	0	0 (0)	1	1 (2.1)
Nausea	1	1 (2.1)	0	0 (0)	1	1 (2.1)

## Discussion

This study successfully completed the first bioequivalence trial of testosterone undecanoate soft capsules in China, filling the gap in pharmacokinetic evaluation and bioequivalence verification of this oral formulation in the Chinese population. As a highly variable endogenous hormonal drug, testosterone undecanoate has long faced challenges in bioequivalence studies due to significant intra-participant variability and complex baseline correction requirements. Through the implementation of a four-period fully replicated crossover design and reference-scaled average bioequivalence (RSABE) methodology, this research not only validated the bioequivalence between the test and reference formulations but also established a methodological paradigm for bioequivalence studies of highly variable drugs in China. The findings hold critical implications for promoting the market availability of domestic generic drugs and improving medication accessibility for male hypogonadism patients.

The results demonstrated consistency with an international clinical study of the original formulation. This provided justification for selecting healthy postmenopausal women as study participants: pharmacokinetic studies of the original formulation (Andriol Testocaps^®^) in postmenopausal women showed plasma testosterone C_max_ of 3.86 ± 1.99 ng/mL (N = 15) following high-fat meal administration of 40 mg.^13^ In this trial, unadjusted plasma testosterone C_max_ values for the test and reference formulations (Andriol Testocaps^®^) under postprandial conditions were 4.03 ± 2.11 ng/mL (N = 96) and 3.89 ± 1.80 ng/mL (N = 96), respectively, closely aligning with literature data and confirming the validity of participant screening criteria and dosing conditions while preliminarily verifying consistency in key exposure metrics between formulations.

Furthermore, multiple international studies confirmed the safety and tolerability of the original formulation in postmenopausal women, with no severe adverse events reported (Houwing et al., 2003; Schnabel et al., 2007; Bagchus et al., 2003). This trial exhibited a low adverse event rate (14.6%), all classified as Grade 1 reversible events (e.g., urinary leukocyte positivity), with no serious adverse reactions, consistent with long-term safety data for oral testosterone undecanoate (Honig et al., 2022; Rivero et al., 2024). Notably, the oral soft capsule formulation bypasses hepatic first-pass metabolism via lymphatic absorption, offering advantages in convenience and safety compared to injectable or transdermal formulations (Campbell et al., 2023). Patient satisfaction studies further demonstrated that oral formulations significantly improved treatment adherence (30% increase in TSQM-9 scores), supporting clinical translatability (Rivero et al., 2024; Campbell et al., 2023).

European clinical applications over 3 decades have confirmed the safety and tolerability of oral testosterone undecanoate formulations. However, optimal therapeutic efficacy requires dietary management to enhance bioavailability. Testosterone undecanoate capsules must be administered with lipid-containing meals to facilitate lymphatic absorption; otherwise, serum testosterone levels significantly decline, compromising therapeutic outcomes. This trial strictly adhered to this pharmacological characteristic by implementing a high-fat meal protocol prior to dosing, ensuring accurate assessment of true bioequivalence under optimal absorption conditions (Bagchus et al., 2003; Köhn and Schill, 2003).

The study design rigorously followed the NMPA‘s *Technical Guidelines for Pharmacokinetic Evaluation of Bioequivalence in Chemical Drug Generic Products* (China National Medical Products Administration, 2016) and the FDA’s bioequivalence guidelines (U.S. Food and Drug Administration, 2021) for testosterone undecanoate soft capsules. For testosterone undecanoate, a four-period two-sequence crossover design was employed. To address its endogenous nature, multiple baseline sampling points and corrections were implemented to mitigate interference from endogenous testosterone fluctuations. Blood sampling windows covered absorption, peak concentration, and elimination phases, balancing FDA requirements with literature recommendations (Testosterone Undecanoate-Schering AG, 2004).

In accordance with the NMPA’s *Technical Guidelines for Bioequivalence Studies of Highly Variable Drugs* (China National Medical Products Administration, 2018), this study innovatively applied the RSABE method with dynamic adjustment of equivalence criteria based on reference formulation CV_w_%. With a testosterone undecanoate C_max_ CV_w_% of 50.91%, the adjusted GMR (102.20%) remained within equivalence bounds after RSABE analysis. This approach aligned with FDA recommendations for highly variable drugs and Pastuszak’s population modeling (Pastuszak et al., 2021), demonstrating methodological advancement.

Limitations should be acknowledged: First, the study population comprised healthy postmenopausal women rather than the target patient population (male hypogonadism patients), necessitating future validation in clinical populations. Second, despite the four-period design, the high variability (C_max_ CV_w_% >50%) warrants larger sample sizes or model-informed bioequivalence evaluation (MIBBE) in subsequent studies to enhance statistical power and extrapolation reliability. Third, the open-label design may have introduced potential bias in the reporting and assessment of subjective adverse events, such as nausea.

## Conclusion

This study evaluated the pharmacokinetic profiles of testosterone undecanoate soft capsules (40 mg) and demonstrated that the test formulation and reference formulation exhibited comparable bioequivalence under postprandial conditions in healthy Chinese postmenopausal female participants. Additionally, both formulations were well-tolerated, with no safety concerns identified during the trial.

## Data Availability

The raw data supporting the conclusions of this article will be made available by the authors, without undue reservation.
