# Supplementary material for: Bioequivalence study of testosterone undecanoate soft capsules in healthy postmenopausal women under fed conditions: a single-center, four-period, repeated crossover trial
Source: Front Pharmacol. 2026 Mar 3;17:1763835. doi: 10.3389/fphar.2026.1763835 (PMC12992283; doi:10.3389/fphar.2026.1763835)
Supplement: Supplementary file 1 [file Supplementaryfile1.docx]

**Inclusion Criteria**

1. Provided written informed consent after full understanding of the study objectives, procedures, and potential adverse reactions;
2. Healthy naturally postmenopausal females aged 45-65 years (inclusive), with cessation of menses for ≥12 months;
3. Body weight ≥45.0 kg and body mass index (BMI) between 19.0-28.0 kg/m2 (inclusive);
4. No history of chronic or severe diseases in cardiovascular, hepatic, renal, respiratory, hematologic/lymphatic, endocrine, immune, psychiatric, neurological, or gastrointestinal systems, and in general good health;
5. Normal findings or clinically insignificant abnormalities in physical examination (including vulvar assessment), vital signs, 12-lead electrocardiogram (ECG), laboratory tests [blood biochemistry, hematology, urinalysis, coagulation function, pregnancy test, four infectious disease markers (HBsAg, anti-HCV, anti-HIV, TPAb), sex hormone panel (total testosterone ≤0.75 ng/mL)], alcohol breath test, drug abuse screening, and breast ultrasound;
6. Ability to communicate effectively with investigators and comply with all study requirements.

**Exclusion Criteria**

1. Subjects with a history of allergy to testosterone undecanoate or any component of the formulation, or those with hypersensitivity to ≥2 drugs/food items;
2. Subjects with acne, hirsutism, hoarseness, alopecia, myocardial infarction, or unstable cardiovascular diseases;
3. Postmenopausal females with unexplained vaginal bleeding;
4. Lactose-intolerant individuals (manifested by diarrhea after milk consumption);
5. Subjects with dysphagia or gastrointestinal disorders affecting drug absorption;
6. Individuals intolerant to venipuncture or with a history of needle phobia;
7. Subjects undergoing surgery within 3 months prior to screening or planned surgical interventions during the study, or those receiving procedures impacting drug absorption/distribution/metabolism/excretion;
8. Subjects donating blood (including components) or experiencing blood loss ≥400 mL within 3 months prior to screening, or receiving blood transfusions/blood products;
9. Subjects with a history of drug abuse within 6 months prior to screening;
10. Illicit drug use within 3 months prior to screening;
11. Administration of any hepatic enzyme-inducing (e.g., barbiturates, carbamazepine, phenytoin, glucocorticoids, omeprazole) or inhibiting (e.g., SSRIs, cimetidine, diltiazem, macrolides, nitroimidazoles, sedatives, verapamil, fluoroquinolones, antihistamines) medications within 4 weeks prior to investigational product administration, or concomitant use of interacting drugs (e.g., coumarins, corticosteroids, estrogen/progesterone);
12. Vaccination within 4 weeks prior to investigational product administration or planned vaccinations during the study;
13. Use of prescription/non-prescription medications, herbal products, or vitamins within 2 weeks prior to investigational product administration, as deemed clinically significant by the investigator;
14. Daily tobacco consumption >5 cigarettes within 3 months prior to screening, or inability to abstain during the study;
15. Weekly alcohol intake >14 units (1 unit ≈360 mL beer/45 mL 40% spirit/150 mL wine) within 3 months prior to screening, or inability to abstain;
16. Daily intake of >8 cups (1 cup=250 mL) of tea/coffee/caffeinated beverages within 3 months prior to screening, or unwillingness to restrict intake;
17. Consumption of dragon fruit, mango, grapefruit, lime, starfruit, or related products within 7 days prior to screening, as assessed by the investigator;
18. Subjects requiring special diets incompatible with the standardized protocol;
19. Participation in another clinical trial involving investigational drugs within 3 months prior to administration, or non-compliant attendance;
20. Consumption of chocolate, caffeine-containing, or xanthine-rich foods/drinks between screening and Day -1, as deemed interfering;
21. Development of acute illnesses prior to investigational product administration;
22. Any other condition deemed unsuitable for participation by the investigator.

**Supplementary Materials**

Supplementary table. 1. Mean pharmacokinetic parameters (PKPS) (baseline-corrected testosterone)

|  | **T** | **R** |
| --- | --- | --- |
| T_max_^#^（h） | 7.49（1.9917,13.9917）（N=96） | 6.99（2.9917,12.9922）（N=96） |
| C_max_（ng/mL） | 3.92±2.11（53.74）（N=96） | 3.78±1.79（47.42）（N=96） |
| AUC_0-t_（h*ng/mL） | 22.26±8.03（36.08）（N=96） | 21.95±6.97（31.74）（N=96） |
| AUC_0-∞_（h*ng/mL） | 22.82±8.06（35.34）（N=95） | 22.46±6.98（31.07）（N=96） |
| λ_Z_（1/h） | 0.2190±0.0602（27.51）（N=95） | 0.2201±0.0659（29.95）（N=96） |
| t_1/2_（h） | 3.49±1.39（39.87）（N=95） | 3.49±1.34（38.43）（N=96） |
| AUC__%Extrap（%）_ | 2.55±2.17（84.93）（N=95） | 2.41±1.83（75.77）（N=96） |

Note: T_max_ is presented as median (minimum, maximum). For Subject C028 (dosing sequence T-R-T-R) in Period 3, fewer than 3 non-below-the-limit-of-quantification (non-BQL) sampling points were available following testosterone Cmax. Consequently, lambda_z (λ_z_), t_₁/₂_, AUC_₀₋∞_ (area under the curve extrapolated to infinity), and AUC__%Extrap_ (percentage of AUC extrapolated beyond the last measurable concentration) for the corresponding period were treated as missing data.

Supplementary table. 2. Mean pharmacokinetic parameters (PKPS) (non-baseline-corrected testosterone)

|  | **T** | **R** |
| --- | --- | --- |
| T_max_^#^（h） | 7.49（1.9917,13.9917）（N=96） | 6.99（2.9917,12.9922）（N=96） |
| C_max_（ng/mL） | 4.03±2.11（52.40）（N=96） | 3.89±1.80（46.33）（N=96） |
| AUC_0-t_（h*ng/mL） | 24.89±8.28（33.25）（N=96） | 24.52±7.33（29.90）（N=96） |
| AUC_0-∞_（h*ng/mL） | 26.62±8.32（31.26）（N=95） | 26.13±7.42（28.40）（N=96） |
| λ_Z_（1/h） | 0.1479±0.0489（33.05）（N=95） | 0.1487±0.0493（33.12）（N=96） |
| t_1/2_（h） | 5.50±2.89（52.47）（N=95） | 5.43±2.97（54.62）（N=96） |
| AUC__%Extrap（%）_ | 6.78±4.34（64.02）（N=95） | 6.40±4.21（65.78）（N=96） |

Note: T_max_ is presented as median (minimum, maximum). For Subject C028 (dosing sequence T-R-T-R) in Period 3, fewer than 3 non-below-the-limit-of-quantification (non-BQL) sampling points were available following testosterone Cmax. Consequently, lambda_z (λ_z_), t_₁/₂_, AUC_₀₋∞_ (area under the curve extrapolated to infinity), and AUC__%Extrap_ (percentage of AUC extrapolated beyond the last measurable concentration) for the corresponding period were treated as missing data.

Supplementary table. 3. Testosterone undecanoate sensitivity analysis: AUC₀₋∞ bioequivalence analysis (BES) (testosterone undecanoate) (excluding AUC_%Extrap > 20%)

|  | **ABE** | | | | | | **RSABE** | | | | | |
| --- | --- | --- | --- | --- | --- | --- | --- | --- | --- | --- | --- | --- |
|  | **N_T_** | **GLSM （T）** | **N_R_** | **GLSM （R）** | **GLSMR （T/R）%** | **90%CI（%）** | **S_WR_** | **CV_w_（%）** | **acceptance criterion** | **T/R ratio**  **(%)** | **Power%** | **Application methods** |
| AUC_0-∞_（h*ng/mL） | 93 | 259.00 | 93 | 260.89 | 99.28 | 92.27-106.81 | 0.2900 | 29.62 | -0.0491 | 100.12 | 99.92 | ABE |

**Note:** The acceptance criterion was defined as the upper bound of the one-sided 95% confidence interval for ${\text{（}\text{}\text{Y}_{\text{T}}\text{−}{\text{}\text{Y}}_{\text{R}}\text{）}}^{\text{2}}\text{−}\text{θ}\text{S}_{\text{WR}}^{\text{2}}$. For Subject C010 (dosing sequence R-T-R-T) in Period 1 and Subject C028 (dosing sequence T-R-T-R) in Period 3, fewer than 3 non-below-the-limit-of-quantification (non-BQL) sampling points were available following testosterone undecanoate Cmax. Consequently, AUC₀₋∞ (area under the curve extrapolated to infinity) for the corresponding periods was treated as missing data. For Subjects C007 (Period 1, dosing sequence T-R-T-R), C008 (Periods 1 and 2, dosing sequence T-R-T-R), and C033 (Period 2, dosing sequence T-R-T-R) where AUC_%Extrap > 20%, the corresponding period’s AUC₀₋∞ was excluded to perform equivalence sensitivity analysis.

Supplementary table. 4. Bioequivalence analysis (BES) (baseline-corrected testosterone)

|  | **ABE** | | | | | | **RSABE** | | | | | |
| --- | --- | --- | --- | --- | --- | --- | --- | --- | --- | --- | --- | --- |
|  | **N_T_** | **GLSM （T）** | **N_R_** | **GLSM （R）** | **GLSMR （T/R）%** | **90%CI（%）** | **S_WR_** | **CV_w_（%）** | **acceptance criterion** | **T/R ratio**  **(%)** | **Power%** | **Application methods** |
| C_max_（ng/mL） | 96 | 3.46 | 96 | 3.42 | 101.31 | 93.94-109.26 | 0.3145 | 32.24 | -0.0576 | 101.31 | >99.99 | RSABE |
| AUC_0-t_（h*ng/mL） | 96 | 20.89 | 96 | 20.94 | 99.75 | 95.44-104.27 | 0.1701 | 17.14 | -0.0170 | 99.75 | >99.99 | ABE |
| AUC_0-∞_（h*ng/mL） | 95 | 21.44 | 96 | 21.46 | 99.91 | 95.61-104.41 | 0.1716 | 17.29 | -0.0174 | 100.08 | >99.99 | ABE |

Note: The acceptance criterion was defined as the upper bound of the one-sided 95% confidence interval for ${\text{（}\text{}\text{Y}_{\text{T}}\text{−}{\text{}\text{Y}}_{\text{R}}\text{）}}^{\text{2}}\text{−}\text{θ}\text{S}_{\text{WR}}^{\text{2}}$. For Subject C028 (dosing sequence T-R-T-R) in Period 3, fewer than 3 non-below-the-limit-of-quantification (non-BQL) sampling points were available following testosterone Cmax. Consequently, AUC₀₋∞ (area under the curve extrapolated to infinity) for the corresponding period was treated as missing data.

Supplementary table. 5. Bioequivalence analysis (BES) (non-baseline-corrected testosterone)

|  | **ABE** | | | | | | **RSABE** | | | | | |
| --- | --- | --- | --- | --- | --- | --- | --- | --- | --- | --- | --- | --- |
|  | **N_T_** | **GLSM （T）** | **N_R_** | **GLSM （R）** | **GLSMR （T/R）%** | **90%CI（%）** | **S_WR_** | **CV_w_（%）** | **acceptance criterion** | **T/R ratio**  **(%)** | **Power%** | **Application methods** |
| C_max_（ng/mL） | 96 | 3.59 | 96 | 3.53 | 101.48 | 94.35-109.15 | 0.3059 | 31.32 | -0.0544 | 101.48 | >99.99 | RSABE |
| AUC_0-t_（h*ng/mL） | 96 | 23.60 | 96 | 23.51 | 100.38 | 96.54-104.38 | 0.1558 | 15.68 | -0.0143 | 100.38 | >99.99 | ABE |
| AUC_0-∞_（h*ng/mL） | 95 | 25.35 | 96 | 25.14 | 100.84 | 97.09-104.72 | 0.1571 | 15.81 | -0.0143 | 100.95 | >99.99 | ABE |

Note: The acceptance criterion was defined as the upper bound of the one-sided 95% confidence interval for ${\text{（}\text{}\text{Y}_{\text{T}}\text{−}{\text{}\text{Y}}_{\text{R}}\text{）}}^{\text{2}}\text{−}\text{θ}\text{S}_{\text{WR}}^{\text{2}}$. For Subject C028 (dosing sequence T-R-T-R) in Period 3, fewer than 3 non-below-the-limit-of-quantification (non-BQL) sampling points were available following testosterone Cmax. Consequently, AUC₀₋∞ (area under the curve extrapolated to infinity) for the corresponding period was treated as missing data.
